# A comparative study of Korean and Chinese learners’ strategy use in request-refusal continuous dialogue speech acts

**DOI:** 10.1371/journal.pone.0354888

**Published:** 2026-07-30

**Authors:** Liang Xu, Xiao Wu

**Affiliations:** 1 School of Resources and Environment, Qingdao Agricultural University, Qingdao, China; 2 International Office of Cooperation & Exchange, Qingdao Agricultural University, Qingdao, China; Xi’an Jiaotong-Liverpool University, CHINA

## Abstract

In daily communications, the use of speech acts that may threaten others’ faces, such as request and refusal, is inevitable. By methods of DCT with 24 coherent request-refusal scenarios as well as optimized classification of request and refusal strategies, differences in request-refusal continuous dialogue speech acts between Native Korean (NK) and Chinese Korean language learners of intermediate level (CKLI) and advanced level (CKLA) were examined. There were statistically significant differences in frequencies of NK, CKLA, and CKLI using request and refusal strategies in different scenarios. Overall, NK used more categories and numbers of strategies than CKLI. The gap between NK and CKLI might narrow as language proficiency increases. Furthermore, not only the single influence factor that affected strategy choice but also the interactions between these factors, suggesting that people might behave differently when faced with complex scenarios consisting of multiple factors. This study also proposes a new research methodology that inserts factors affecting strategy choice into individual scenarios, designs multiple independent scenarios with the same factors, and analyses the effects of these factors and their interactions on strategy choice. The results of the associated analysis could be used to assist in the development of a detailed instructional plan.

## Introduction

China and South Korea are two geographically close neighbors. The two countries have in-depth exchanges and cooperation in various fields such as economy and trade, politics, humanities, science and technology, etc. There is a great demand for Korean language talents in China, especially in Shandong Province. Compared with other provinces, Shandong Province has unique geographical and cultural advantages. The large-scale investment of Korean companies in Shandong and the large amount of trade with Korea make Shandong the most favorable location for Korean investment in China. Many universities and vocational schools in China offer majors and related courses in Applied Korean.

Whatever the motivation, one of the purposes of learning a language is to be able to communicate flexibly with interlocutors in that language. In this process, the speaker’s assertions, demands, questions, information provision, and other general communicative acts, as well as the listener’s understanding and response, are interactive.

Face Threatening ACT (FTA in short) often inevitably occurs when people are interacting with each other, especially in the face of requests or refusals from others. As an essential social activity in interpersonal communication, requesting behavior is very sensitive and highly used in daily life. The request is considered a type of FTA because it is usually self-serving and requires time, effort, or material cost to the requested person [1]. The speech act of refusal is also regarded as a type of FTA because of its disobedient nature [2]. In refusing a directive discourse (e.g., request, suggestion), the speaker threatens his or her negative image; while in refusing a commanding discourse (e.g., offer, invitation), the speaker refuses to support his or her positive image. Once refused, the self-esteem of both the speaker and the listener can be potentially jeopardized, impede social interactions, or even cause offense. It is unpleasant for anyone to refuse another person’s request or to be refused by another person under any circumstances and for any reason. This is because refusal negates the essential attributes of another person’s speech act, creates an awkward situation, and inevitably threatens or undermines the dignity of the interlocutor. For this reason, the speech acts of refusal are sometimes perceived as insulting.

When implementing an FTA (e.g., refusal or request), speakers must consider sociolinguistic variables such as closeness, age, gender, and social status to choose the appropriate speaking strategy that best accomplishes their goals and avoids damaging the other party’s face. In this process, cultural factors influence the speaker’s choice of content and strategy no less than language. Contextual dependence on cultural and social situations is an important factor in determining the success of communication.

South Korea and China belong to the same East Asian cultural circle with a long history of exchanges. As Korean culture has been deeply influenced by Confucianism, the two countries share certain cultural commonalities. In recent times, Korea has been influenced by other countries such as the United States and Japan in many ways, resulting in differences in living environment, values, and ways of thinking. As a result, both Korea and China now have their cultural characteristics in terms of language and non-language. When communication difficulties occur, native speakers are more likely to judge the foreign learner as rude and uncooperative, rather than finding the cause in the deficiencies of the foreign learner’s language resources [3]. Another study on Koreans’ reactions to foreigners’ refusal behaviors has found that Koreans reacted more negatively to content issues than to grammatical issues, which is closely related to Korean culture, social habits, and so on [4]. Therefore, to prevent and resolve communicative conflicts caused by cultural differences, it is necessary to conduct an in-depth comparative analysis of Korean linguistic and cultural studies for learners of the dominant culture to find out the scenarios and causes of conflicts and to reflect them in the subsequent teaching process.

## Literature review

### Prior studies on the speech act of request

The request is a speech act that falls under Searle’s category of “instruction” and is an attempt by the speaker to get the hearer to do something, which may be a very gentle attempt or a very vigorous one” [5].

There have been many comparative studies on the speech act of request in Korean and other languages, or between Koreans and people from other countries, including English [6–8], Japanese [9], Thai [10], French [11], Chinese [12], and so on.

Many comparative studies on politeness expression between languages have found that different cultural differences are important for politeness expression, since cultural misunderstandings may lead to miscommunication or even communication failure, polite expression becomes very important. Besides, expressing politeness in a second language affects one’s politeness expression.

Compared to grammatical errors, Koreans were less aware of discourse violations and had speech act strategies that deviated from those of native English speakers. In terms of request speech acts, Koreans rely on politeness markers rather than the negative politeness strategies preferred by native English speakers [6]. Differences in politeness between American and Korean cultures, such as strategic politeness and discriminative politeness, lead to different patterns of polite behavior in terms of request strategy choices [8]. Politeness is perceived differently in different cultures. For example, neither unconventional indirectness nor certain conventional indirectness strategies in the Korean language implied politeness. In the request category, conventional indirect strategies (e.g., strong Hints, mild Hints, and suggestive formulate) were not statistically significantly associated with politeness in Korean, whereas “performatives” and “want statements” were perceived as direct but polite strategies. These results demonstrate that there are statistically significant differences in the degree and conceptualization of politeness in Korean, Hebrew, and English [7]. In a study comparing common request speech acts in the original Japanese TV program and the Korean remake, it was found that Korea used direct strategies slightly more frequently than Japan, while Japan used indirect strategies slightly more frequently than Korea [9]. Thai learners of Korean use requesting strategies with native speakers of Thai, who both use requesting strategies such as direct strategies, conventional indirect strategies, unconventional indirect strategies, and concealment of intentions. There are no obligatory statements among Korean learners, while Thai native speakers use more internal request modifiers [10]. Being East Asian countries and influenced by Confucianism, China, and South Korea share some similarities with other countries in terms of request speech acts. Language shifts, inductive/deductive ways of thinking, unfamiliarity, and different perceptions of politeness may account for differences in request strategies. Both the Chinese speakers and the South Korean learners tended to use “query preparatory” and “mood derivable” more directly as head acts, while the French learners tended to be more indirect by primarily using “query preparatory.” In terms of socio-pragmatics, both the Korean learners and the Chinese preferred hierarchy and collectivism, while the French students favored egalitarianism and individualism [11]. In a study specifically focusing on Chinese and Korean request speech acts, it was found that Chinese and Koreans used more direct request strategies in natural conversation compared to role-playing, but that Chinese native speakers would make requests more indirectly than Korean native speakers [12].

### Prior studies on the speech act of refusal

Refusal is a response speech act in which the respondent refuses to participate in the action proposed by the interlocutor [13,14], which has been described as a major cross-cultural barrier for many non-native speakers [15]. Because of the face-threatening nature of refusal, it usually requires a lengthy negotiation process, the form and content of which varies according to the eliciting speech act. Beebe et al. (1990) categorized refusal strategies and is one of the most widely used refusal taxonomies.

Several studies have been conducted on comparisons of refusal speech acts between Korean and other languages or between Koreans and people from other countries, e.g., English [16], Egyptian [17], Korean Expressions for South and North Korea [18], Japanese, Indonesian, Vietnamese, Filipino [19], Chinese [20–22].

A study involving Korean and Norwegian English learners of English found statistically significant differences in the use of direct and indirect strategies, but the effect sizes were small. The difference in the use of adjunct strategies between the two groups was not statistically significant. The findings suggest that the pragmatic performance of multilinguals is a complex phenomenon that cannot be explained solely by differences in cultural and pragmatic norms of the native language [16]. The refusal strategies used by Egyptians learning Korean were sometimes similar to Egyptian refusal styles and sometimes similar to Korean refusal styles, suggesting that the refusal styles of Egyptians learning Korean were characterized by “Interlanguage” [17]. Even when similar languages are used, there are statistically significant differences between refusal strategies. North Korean refugees preferred direct verbal acts of rejection to indirect verbal acts of rejection, and this preference varied according to the type of verbal act induced. North Korean refugees and South Koreans differed in their personality evaluations of the same refusers, and this difference might lead to miscommunication. North Korean refugees’ evaluations of refusal speech acts did not change over time. South Koreans and North Koreans reacted differently to expressions used only in North Korea. As a result, not only refusal strategies, but refusal expressions themselves are important for successful communication. Refusal made for non-essential and non-personal reasons were more favorable than those made for personal reasons. These findings suggest that pragmatic awareness in language use is helpful for successful communication between two groups with different linguistic backgrounds [18]. The refusal expressions in six Asian languages, including Japanese, Indonesian, Korean, Vietnamese, Filipino, and Chinese, were investigated to examine whether there was a relationship between language category (head-initial/final language) and the occurrence of semantic formula function groups in refusal expressions. The results of the analysis of refusal expressions of the six languages showed that head-initial languages have a strong tendency to use “Refusal” before another semantic formula, and head-final languages have a tendency to use “Refusal” after other semantic formula function groups. The respondents also change their way of refusing depending on their closeness to the requester [19]. In the comparative studies of the refusal strategies of Chinese learners of the Korean language and native Koreans, it was found that Koreans mostly chose to refuse others’ requests or suggestions indirectly, and only about 7% of the Koreans interviewed chose to refuse directly in different scenarios. The effect of indirect refusal is based on the analysis of the context and the judgment of the intention, which helps to reduce the burden of the refusal conversation to some extent. Korean and Chinese learners usually employed more than one strategy for refusal. There was a statistically significant difference in the frequency of use of refusal strategies by Chinese learners with different language proficiency and native Korean in different scenarios, and this gap narrowed as language proficiency improved [13,22]. In the workplace, Korean native speakers used more indirect strategies + direct strategies than other strategies, while Chinese native speakers used more indirect strategies than others. When Chinese native speakers refused requests from bosses or coworkers, they got less psychological pressure than Korean native speakers. In addition, Korean native speakers and Chinese native speakers used different linguistic expressions when refusing work-related requests [20].

### Limitations of prior studies

Most request strategy research draws on the strategy categorization methodology and research methods of classic work [1,23]. The unit of analysis for request acts in the DCT is the utterance or sequence of utterances supplied by the informant in completing the test item. In the study procedure, the request speech act could be analyzed with the following segments: (a) Address Term(s); (b) Head Act; (c) Adjunct(s) to Head Act. However, requests are not necessarily made in this order, and the order can change depending on variables. Some studies have analyzed only Head Act alone, which tends to be biased, and it is necessary to study them in conjunction with the entire phase of the request. A very large number of studies have focused on the similarities and differences in requesting and refusing strategies and expressions between groups, while there have been fewer comparative and cognitive studies on intergroup language and culture. Especially the refusal speech act that can easily threaten the face of other people, it is important to carefully choose the appropriate way of refusal in different scenarios, taking into account the status of both parties, age, gender, intimacy, and so on.

In contrast to the large number of cross-cultural comparative studies on request or refusal speech acts between Western languages or between Western and Asian languages [24], relatively few studies have been conducted on Asian languages, such as comparisons between Korean and Chinese [13]. The vast majority of the current research on request or refusal speech acts, including strategies and expressions in Korean and Chinese, has been conducted using the Discourse Completion Test (DCT) by setting up situations with fixed numbers of variables [10,12–14,17]. Many studies have used DCT as the sole means of collecting data. Although DCTs enable large‑sample speech act collection, the data may not fully represent naturalistic interaction, and responses may differ from authentic speech acts in real situations. However, it is unlikely that the variables distributed in each scenario will fully cover all aspects of real life. To achieve the goals, request or refusal speech acts often use multiple strategies, which may be of different types, and the request or refusal act may go through multiple rounds. Yet many DCT studies simply ask respondents to respond based on set scenarios. In addition, some DCT scenarios appear to be quite unrealistic [25], and some studies have limited sample sizes, all of which may have had a statistically significant impact on the results of the study. Some of the studies that did not employ DCT used linguistic materials such as television dramas, television talk shows, literature, and textbooks, which are susceptible to subjective authorial ideas, acquired linguistic formulas, and the unnatural style of television programs [9,13].

Therefore, further study is needed on Korean request and refusal speech acts between Chinese learners and Korean native speakers. Request and refusal speech acts tend to occur simultaneously in a conversational scenario, and the two speech acts should not be studied separately and in isolation. The study should be based on a reasonable setting of DCT investigation scenarios, collecting and summarizing closer to real conversation data from multiple similar scenarios and multiple rounds of conversations, so that the results can more realistically reflect the changing characteristics of speech acts.

## Methodology

### Context and participants

In this study, the DCT survey method was used for data collection and analysis by answering questionnaires and dialogues. A preliminary survey was conducted from March to June 2018 using designed questionnaires with current students (Korean majors and Korean learners) at several universities located in Qingdao, China, as well as Korean study abroad students in Qingdao [21]. Chinese learners were categorized by levels into intermediate (passing TOPIK level 3–4, hereinafter CKLI) and advanced (passing TOPIK level 5–6, hereinafter CKLA). Native Korean students were hereinafter referred to as NK. Some participants in the three surveyed groups (i.e., NK, CKLA, and CKLI) noted that some of the request-refusal dialogue scenarios in the questionnaire were difficult to encounter in real life. In particular, some of the request scenarios did not reflect the characteristics of real-life dialogues because only simple request strategies can be used. With the help of the participants, the questionnaire was improved to make the request and refusal scenarios more relevant to daily life to eliminate interference in the subjects’ strategy choices. Based on the data from the new questionnaire, we analyzed the characteristics of request and refusal acts and the relationship between request and refusal pairs. A questionnaire study was conducted from March to July 2019, and from June to December 2023 with students from several colleges and universities in Qingdao and South Korea, with the total number of participating NKs, CKLAs, and CKLIs being 43, 67, and 84, respectively.

This study does not fall into the categories that require mandatory ethical approval, including human/animal physiological or psychological interventions, clinical diagnosis and treatment, invasive procedures, and biological sample collection. Classified as a non-interventional, low-risk research project, it therefore does not necessitate the acquisition of ethical approval. This research was conducted via face-to-face administration of a pre-designed questionnaire. All participants in the study took part voluntarily; prior to the investigation, they were fully informed of the research objectives, data usage purposes, and their right to withdraw from the study. The entire research process strictly adhered to the general academic norms in the field of linguistics.

### Study design

Many factors may influence the use of strategies in request and refusal speech acts. In this study, three variables, social status, the intimacy of the relationship between the interlocutors, and the difficulty of the speech act, were selected to study the effect on communication strategies. Social status was subdivided into “equal” and “unequal”, intimacy was subdivided into “intimate” and “not intimate”, and difficulty was subdivided into “easy” and “difficult”. Each variable was cross-combined with each other to produce eight combinations. To reduce error, the ANOVA results should be representative and generalizable, so we set up three replicate scenarios for each combination, for a total of 24 scenarios (Table 1). The level of social status was determined by the status of the speaker’s counterpart, i.e., the listener. According to the general theory, bosses, elders, senior people and people with high social duties tend to have high status. Intimacy referred to the degree of familiarity between the two parties to the conversation. Difficulty refers to the perceived pressure experienced by the requester or the refuser. It is high when the requester imposes great pressure on the hearer, or when the refuser experiences high pressure themselves. The level of burden is subjectively judged by the researcher based on experience. The level of difficulty of the questions was adjusted based on preliminary findings and participant feedback. Based on previous studies, request and refusal strategies were classified into seven categories respectively (Table 2), with which the data were analyzed [10,13–15,23,26].

**Table 1:** Eight combinations of request scenarios and eight combinations of refusal scenarios.

Speech act	Combination	Variables		
		Social status	Intimacy	Difficulty
Request	1	Equal	Intimate	Easy
	2	Equal	Not intimate	Easy
	3	Equal	Intimate	Difficult
	4	Equal	Not intimate	Difficult
	5	Unequal	Intimate	Easy
	6	Unequal	Not intimate	Easy
	7	Unequal	Intimate	Difficult
	8	Unequal	Not intimate	Difficult
Refusal	1	Equal	Intimate	Easy
	2	Equal	Not intimate	Easy
	3	Equal	Intimate	Difficult
	4	Equal	Not intimate	Difficult
	5	Unequal	Intimate	Easy
	6	Unequal	Not intimate	Easy
	7	Unequal	Intimate	Difficult
	8	Unequal	Not intimate	Difficult

**Table 2:** Framework for analyzing request and refusal discourses in this study.

Number	Strategy categories of request	Strategy categories of refusal Major category name (the name of the included strategy)
1	Direct request	Direct refusal
2	Mention conditions	Provide a reason or rationale
3	Ask for information	Avoidance (Change the topic/avoidance / make non-verbal statements)
4	Statement of obligation	Conditional acceptance (Make a suggestion / conditional acceptance)
5	Make a suggestion	Subjective causes (assertion / self-blame)
6	Express a wish	Accusation (Accusation/question)
7	Statement of situation	Express emotions (apologize/thank/congratulate)

This research design was adopted because there is little research on the interactive effects of various factors that influence communication behavior. Therefore, we proposed this method for studying the effects of multiple factors and their interactions on interrelated speech acts and named this method “Methodology Based on Interaction and Combination of Multi-factors (hereinafter referred to as MICMF)”. This method designs multiple independent scenarios with the same factors by inserting each factor affecting strategy choice in each scenario, combining them, and analyzing the effects of the factors and their interactions on strategy choice. This study aims to identify the differences in strategy choice between native Korean and Chinese learners in different contexts by analyzing multifactor cross-fertilization language scenarios and to help develop a detailed teaching plan based on the results of the analysis. The detailed protocol documents are publicly available at protocols.io and can be accessed via the following DOI: https://dx.doi.org/10.17504/protocols.io.4r3l2djwpg1y/v1.

### Data analysis methods

A dedicated explanation of the definitions for strategy statistics is provided below. The “Strategy category” refers to the number of types of request or refusal strategies used by participants within the same continuous dialogue scenario (classification criteria see Table 2). The “Strategy number” refers to the total occurrence frequency of all request or refusal strategies used by participants within the same continuous dialogue scenario. For example, if the speaker uses request strategy 3 “Ask for information” twice and request strategy 6 “Express a wish” once in a continuous dialogue, resulting in a statistical value of 2 for request strategy category and a value of 3 for request strategy number. The classification and statistical approach can accurately and separately characterize the diversity of participants’ pragmatic strategy selection and the overall use frequency of their pragmatic strategies. Strategy 1 through Strategy 7 refers to the proportion of number of each specific type of request and refusal strategy established in this study (presented in Table 2), relative to the total number of strategies used.

One-way ANOVA and post-hoc comparisons were used to examine the differences between NK, CKLA, and CKLI in the category and number of request-refusal discourse strategies. In addition, a multifactor ANOVA was used in this study to examine the effects of target people (P), social status (S), intimacy of the relationship (R), difficulty (D), and their interaction on speech acts. All statistical analyses were performed using SPSS 21 (IBM Inc., Armonk, New York, USA). The statistically significant difference was indicated by *p* < 0.05. Origin (Originlab Corporation, Massachusetts, the USA) was used for figure plotting.

## Results

### Request speech acts and associated strategies

Following the effect size reporting and interpretation framework proposed by previous studies [26–28], both statistical significance and substantive effect size values for all analyses are reported in this section, allowing for a quantitative assessment of the substantive magnitude of pragmatic gaps between groups, rather than relying solely on dichotomous “significant *vs.* non-significant.”

**Fig 1:**
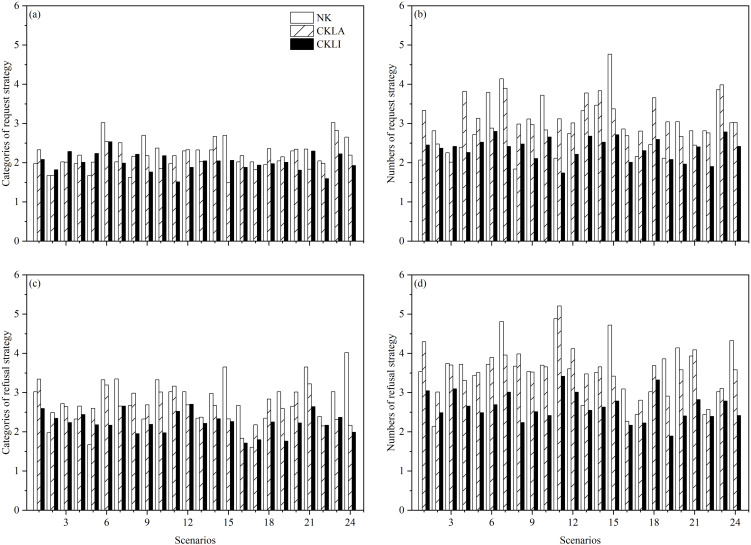
Distribution of request and refusal speech act strategy categories and numbers.

Results of the one-way ANOVA and post-hoc comparisons revealed that the overall between-group difference in the number of request strategy categories used did not reach statistical significance (*p* > 0.05, *eta²* = 0.0698, medium effect). However, post-hoc pairwise comparisons showed that NK speakers used a significantly greater variety of request strategies relative to CKLI (*p* = 0.0315), with a Cohen’s *d* of 0.6278 corresponding to a medium effect. This finding indicates that the difference in the diversity of request strategy use between the two groups has substantive pragmatic meaning. There was no statistically significant difference in the number of request strategy categories between NK and CKLA (*p* = 0.5631, Cohen’s *d* = 0.1511, negligible effect), nor between CKLA and CKLI (*p* = 0.1109, Cohen’s *d* = 0.5389, medium effect) (Table 3; Fig 1).

**Table 3:** Results of one-way ANOVA and post-hoc comparisons for between-group differences in request and refusal strategy use among NK, CKLA and CKLI.

Items of analysis	Request				Refusal			
	*F* and (*eta*^*2*^) value from one-way ANOVA	*P* value from post-hoc comparisons (Cohen’s *d*)			*F* and (*eta*^*2*^) value from one-way ANOVA	*P* value from post-hoc comparisons (Cohen’s *d*)		
		NK-CKLA	NK-CKLI	CKLA-CKLI		NK-CKLA	NK-CKLI	CKLA-CKLI
Strategy category	2.5895 ^ns^ (0.0698)	0.5631 (0.1511)	0.0315 (0.6278)	0.1109 (0.5389)	10.2336 ^***^ (0.2288)	0.2853 (0.2718)	0.0000 (1.1843)	0.0017 (1.2480)
Strategy number	12.1080 ^***^ (0.2598)	0.2796 (−0.2695)	0.0006 (1.0154)	0.0000 (1.7766)	19.5356 ^***^ (0.3615)	0.9280 (0.0230)	0.0000 (1.6089)	0.0000 (1.7987)
Strategy 1	4.2288 ^*^ (0.1092)	0.0514 (0.5439)	0.0060 (0.7832)	0.3975 (0.2745)	1.1412 ^ns^ (0.0320)	0.1589 (0.3674)	0.2546 (0.2974)	0.7839 (0.1119)
Strategy 2	1.3650 ^ns^ (0.0381)	0.1098 (0.4916)	0.2791 (0.3301)	0.5983 (−0.1404)	8.8157 ^***^ (0.2035)	0.0149 (0.7265)	0.0001 (1.1954)	0.0983 (0.4835)
Strategy 3	4.0367 ^*^ (0.1047)	0.1655 (0.3621)	0.0059 (0.7800)	0.1545 (0.5174)	7.9409 ^***^ (0.1871)	0.0007 (0.9021)	0.0013 (0.8234)	0.8438 (0.0987)
Strategy 4	0.9837 ^ns^ (0.0277)	0.2909 (0.3038)	0.1901 (0.3855)	0.7965 (0.0749)	0.4980 ^ns^ (0.0142)	0.3220 (0.2965)	0.5994 (0.1543)	0.6400 (0.1304)
Strategy 5	0.4152 ^ns^ (0.0119)	0.4130 (0.2724)	0.9411 (0.0204)	0.4561 (−0.2030)	0.5816 ^ns^ (0.0166)	0.7684 (0.0854)	0.4555 (0.2054)	0.2992 (0.3201)
Strategy 6	0.9521 ^ns^ (0.0269)	0.6238 (−0.1531)	0.1774 (−0.3954)	0.3873 (−0.2344)	2.7284 ^ns^ (0.0733)	0.2322 (0.2904)	0.2623 (0.3092)	0.0224 (1.0035)
Strategy 7	26.2708 ^***^ (0.4323)	0.0000 (−1.7534)	0.0000 (−1.8299)	0.9376 (0.0233)	0.0592 ^ns^ (0.0017)	0.7318 (0.0939)	0.8705 (0.0465)	0.8574 (0.0566)

*** indicates *P* < 0.001, ** indicates *P* < 0.01, * indicates *P* < 0.05, and ^ns^ indicates no statistically significant difference. *Eta*^*2*^ and Cohen’s *d* values are given in parentheses. Explanation of the definitions for strategy statistics can be found in Data analysis methods.

For the total number of request strategies used in a single dialogue, the overall between-group difference was statistically highly significant (*p* < 0.001, *eta²* = 0.2598, large effect). Post-hoc tests confirmed that NK used a significantly greater number of request strategies than CKLI (*p* = 0.0006), with a Cohen’s *d* of 1.0154 representing a large effect, demonstrating a substantial and meaningful pragmatic gap in the frequency of request strategy use between native speakers and intermediate learners. CKLA also used a significantly greater number of request strategies than CKLI (*p* < 0.0001, Cohen’s *d* = 1.7766, large effect), while there was no statistically significant difference between NK and CKLA (*p* = 0.2796, Cohen’s *d* = −0.2695, small effect) (Table 3; Fig 1).

**Fig 2:**
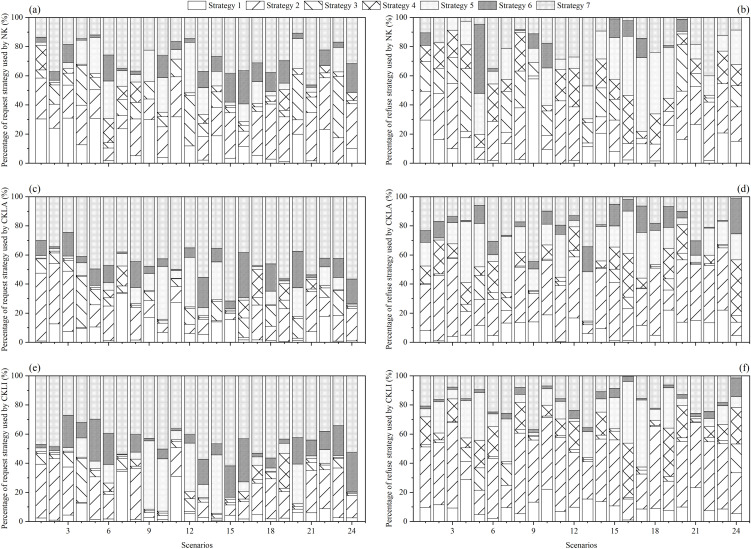
Percentage of different request and refusal strategies used by different groups.

In terms of the proportional use of specific request strategies, there was a statistically significant overall between-group difference in the use of direct request (Strategy 1) (*p* < 0.05, *eta²* = 0.1092, medium effect). Post-hoc comparisons showed that NK used direct request strategies at a significantly higher rate than CKLI (*p* = 0.0060, Cohen’s *d* = 0.7832, approaching a large effect), indicating that this strategy is a key marker distinguishing the pragmatic features of native speakers from intermediate learners. No statistically significant differences were found between NK and CKLA, or between CKLA and CKLI for this strategy (Table 3; Fig 2).

A statistically significant overall between-group difference was also observed in the use of ask for information (Strategy 3) (*p* < 0.05, *eta²* = 0.1047, medium effect). NK used this strategy at a significantly higher percentage than CKLI (*p* = 0.0059, Cohen’s *d* = 0.7800, approaching a large effect), representing another core pragmatic difference between the two groups. No other pairwise comparisons reached statistical significance for this strategy (Table 3; Fig 2).

For the use of statement of the situation (Strategy 7), the overall between-group difference was statistically highly significant (*p* < 0.001, *eta²* = 0.4323, large effect). Post-hoc tests revealed that CKLA used this strategy at a significantly higher rate than NK (*p* < 0.0001, Cohen’s *d* = −1.7534, large effect), and CKLI also used it at a significantly higher rate than NK (*p* < 0.0001, Cohen’s *d* = −1.8299, large effect). There was no statistically significant difference between CKLA and CKLI (*p* = 0.9376, Cohen’s *d* = 0.0233, negligible effect) (Table 3; Fig 2).

No statistically significant overall between-group differences were found for the use of request Strategies 2, 4, 5, and 6 (all *p* > 0.05), with all corresponding *eta²* values below 0.06 (small effect), indicating that the use of these strategies did not differ in a substantively meaningful way across the three groups (Table 3).

A one-way ANOVA identified statistically significant between-group differences in core dimensions of request-refusal strategy use, and a multifactor ANOVA was further conducted to clarify the effects of different influencing factors and their interactions on the use of politeness expression strategies, with detailed results presented in Table 4.

**Table 4:** Results of multi-factor ANOVA examining the effects of target people (P), social status (S), intimacy of the relationship (R), difficulty (D), and their interaction on request speech act strategy use.

	Strategy category	Strategy number	Strategy 1	Strategy 2	Strategy 3	Strategy 4	Strategy 5	Strategy 6	Strategy 7
P	2.8299 ^ns^ (0.1055)	14.2482 ^***^ (0.3725)	4.1997 ^*^ (0.1489)	1.3468 ^ns^ (0.0531)	4.4995 ^*^ (0.1579)	1.1154 ^ns^ (0.0444)	0.4069 ^ns^ (0.0167)	0.8747 ^ns^ (0.0352)	29.7324 ^***^ (0.5533)
S	1.8166 ^ns^ (0.0365)	2.1721 ^ns^ (0.0433)	0.6938 ^ns^ (0.0142)	2.2055 ^ns^ (0.0439)	0.0551 ^ns^ (0.0011)	5.2868 ^*^ (0.0992)	1.6817 ^ns^ (0.0339)	0.0232 ^ns^ (0.0005)	5.7326 ^*^ (0.1067)
R	0.6053 ^ns^ (0.0125)	2.2189 ^ns^ (0.0442)	5.4040 ^*^ (0.1012)	4.7538 ^*^ (0.0901)	1.8596 ^ns^ 0.0373	2.6737 ^ns^ (0.0528)	3.2072 ^ns^ (0.0626)	0.5842 ^ns^ (0.0120)	7.9672 ^**^ (0.1424)
D	2.7050 ^ns^ (0.0533)	2.2032 ^ns^ (0.0439)	7.3947 ^**^ (0.1335)	0.0762 ^ns^ (0.0016)	5.3133 ^*^ (0.0997)	3.4965 ^ns^ (0.0679)	0.2389 ^ns^ (0.0050)	4.8958 ^*^ (0.0926)	4.2460 ^*^ (0.0813)
P × S	3.5630 ^*^ (0.1293)	0.5132 ^ns^ (0.0209)	0.4649 ^ns^ (0.0190)	0.8457 ^ns^ (0.0340)	1.8630 ^ns^ (0.0720)	0.7839 ^ns^ (0.0316)	0.2362 ^ns^ (0.0097)	0.0405 ^ns^ (0.0017)	0.6252 ^ns^ (0.0254)
P × R	0.3209 ^ns^ (0.0132)	1.1627 ^ns^ (0.0462)	0.3440 ^ns^ (0.0141)	0.0344 ^ns^ (0.0014)	1.1113 ^ns^ (0.0443)	0.1136 ^ns^ (0.0047)	0.0526 ^ns^ (0.0022)	0.3572 ^ns^ (0.0147)	0.6201 ^ns^ (00252)
P × D	1.9839 ^ns^ (0.0764)	1.9121 ^ns^ (0.0738)	0.5325 ^ns^ (0.0217)	0.4431 ^ns^ (0.0181)	0.2909 ^ns^ (0.0120)	0.7290 ^ns^ (0.0295)	0.0235 ^ns^ (0.0010)	1.9654 ^ns^ (0.0757)	0.9420 ^ns^ (0.0378)
S × R	0.2488 ^ns^ (0.0052)	0.0970 ^ns^ (0.0020)	0.0026 ^ns^ (0.0001)	0.7330 ^ns^ (0.0150)	6.8350 ^*^ (0.1246)	0.0027 ^ns^ (0.0001)	0.9978 ^ns^ (0.0204)	0.9858 ^ns^ (0.0201)	1.7580 ^ns^ (0.0353)
S × D	0.0723 ^ns^ (0.0015)	1.1113 ^ns^ (0.0226)	0.0312 ^ns^ (0.0006)	6.1106 ^*^ (0.1129)	1.1123 ^ns^ (0.0226)	0.0266 ^ns^ (0.0006)	9.5879 ^**^ (0.1665)	1.3180 ^ns^ (0.0267)	0.3858 ^ns^ (0.0080)
R × D	0.0215 ^ns^ (0.0004)	7.1171 ^*^ (0.1291)	2.4488 ^ns^ (0.0485)	1.3916 ^ns^ (0.0282)	1.0297 ^ns^ (0.0210)	7.8985 ^**^ (0.1413)	1.3471 ^ns^ (0.0273)	1.2330 ^ns^ (0.0250)	1.7777 ^ns^ (0.0357)
P × S × R	0.5988 ^ns^ (0.0243)	0.3200 ^ns^ (0.0132)	0.2487 ^ns^ (0.0103)	0.0922 ^ns^ (0.0038)	1.6647 ^ns^ (0.0649)	1.5120 ^ns^ (0.0593)	0.1868 ^ns^ (0.0077)	0.0583 ^ns^ (0.0024)	0.6548 ^ns^ (0.0266)
P × S × D	0.5380 ^ns^ (0.0219)	0.0942 ^ns^ (0.0039)	0.0296 ^ns^ (0.0012)	0.2223 ^ns^ (0.0092)	0.1768 ^ns^ (0.0073)	0.5388 ^ns^ (0.0220)	0.2277 ^ns^ (0.0094)	0.1536 ^ns^ (0.0064)	0.8686 ^ns^ (0.0349)
P × R × D	0.1850 ^ns^ (0.0076)	0.1828 ^ns^ (0.0076)	0.4747 ^ns^ (0.0194)	0.6492 ^ns^ (0.0263)	0.2631 ^ns^ (0.0108)	0.0146 ^ns^ (0.0006)	0.0925 ^ns^ (0.0038)	0.3466 ^ns^ (0.0142)	0.0629 ^ns^ (0.0026)
S × R × D	6.3158 ^*^ (0.1163)	8.6897 ^**^ (0.1533)	0.0729 ^ns^ (0.0015)	0.1930 ^ns^ (0.0040)	1.4313 ^ns^ (0.0290)	0.9428 ^ns^ (0.0193)	0.6875 ^ns^ (0.0141)	0.0007 ^ns^ (0.0000)	0.4376 ^ns^ (0.0090)
P × S × R × D	0.6210 ^ns^ (0.0252)	0.6087 ^ns^ (0.0247)	0.1444 ^ns^ (0.0060)	0.0201 ^ns^ (0.0008)	0.2681 ^ns^ (0.0110)	1.2642 ^ns^ (0.0500)	0.1183 ^ns^ (0.0049)	0.2521 ^ns^ (0.0104)	0.1197 ^ns^ (0.0050)

Groups include NK, CKLA, and CKLI; social status includes equal and unequal; relationship includes intimate and not intimate; difficulty includes easy and difficult. *F* and (*eta*^*2*^) values are given. ^***^ indicates *P* < 0.001, ^**^ indicates *P* < 0.01, ^*^ indicates *P* < 0.05, and ^ns^ indicates no statistically significant difference. Explanation of the definitions for strategy statistics can be found in Data analysis methods.

For the number of request strategy categories used, the main effect of target people (P) did not reach statistical significance (*p* > 0.05, *eta²* = 0.1055, medium effect). The main effects of social status (S), relationship intimacy (R), and difficulty (D) were all non-significant, with corresponding *eta²* values all below 0.05 (small effect), indicating that individual situational factors had limited independent influence on the diversity of request strategy use. Notably, the interaction effect of S × R × D was statistically significant (*p* < 0.05, *eta²* = 0.1163, medium effect). Specifically, the fewest categories of request strategies were used in situations with equal social status, non-intimate relationships, and low task difficulty, while the most categories were used in situations with unequal social status, intimate relationships, and high task difficulty. This result demonstrates that the combination of situational factors exerts a medium, substantive effect on the diversity of learners’ strategy choices. All other interaction effects were non-significant, with corresponding *eta²* values below 0.1 (small effect) (Table 4; Fig 1).

For the total number of request strategies used, the main effect of P was statistically highly significant (*p* < 0.001, *eta²* = 0.3725, large effect), indicating that learners’ language proficiency is the core factor influencing the frequency of request strategy use, with far greater explanatory power than any single situational factor. The interaction effect of R × D was statistically significant (*p* < 0.05, *eta²* = 0.1291, medium effect), with the number of request strategies used by all three groups being relatively high in contexts of intimate relationships and high task difficulty. Meanwhile, the interaction effect of S × R × D was also statistically highly significant (*p* < 0.01, *eta²* = 0.1533, large effect), with the number of request strategies peaking in difficult request scenarios with intimate relationships and unequal social status. This finding indicates that the overlap of multi-dimensional situational factors exerts a large, substantive effect on the frequency of strategy use. All other main effects and interaction effects were non-significant, with corresponding *eta²* values below 0.1 (small effect) (Table 4; Fig 1).

For direct request (Strategy 1), the main effect of P was statistically significant (*p* < 0.05, *eta²* = 0.1489, large effect), as were the main effects of R (*p* < 0.05, *eta²* = 0.1012, medium effect) and D (*p* < 0.01, *eta²* = 0.1335, large effect). Specifically, all three groups used the direct request strategy at a higher rate with intimate interlocutors than with non-intimate ones, and at a higher rate in easy situations than in difficult ones. These two situational factors exerted medium and large substantive effects on the use of this strategy, respectively. All interaction effects were non-significant, with corresponding *eta²* values below 0.05 (small effect) (Table 4; Fig 2).

For mention conditions (Strategy 2), the main effect of R was statistically significant (*p* < 0.05, *eta²* = 0.0901, small-to-medium effect), with all three groups using this strategy at a higher rate with non-intimate interlocutors than with intimate ones. The interaction effect of S × D was also statistically significant (*p* < 0.05, *eta²* = 0.1129, medium effect), with a relatively high rate of use in situations with equal social status and easy requests, and a relatively low rate in situations with unequal social status and easy requests. All other main effects and interaction effects were non-significant, with corresponding *eta²* values below 0.05 (Table 4; Fig 2).

For ask for information (Strategy 3), the main effect of P was statistically significant (*p* < 0.05, *eta²* = 0.1579, large effect), as was the main effect of D (*p* < 0.05, *eta²* = 0.0997, small-to-medium effect). All three groups used this strategy at a higher rate in easy situations than in difficult ones. The interaction effect of S × R was statistically significant (*p* < 0.05, *eta²* = 0.1246, medium effect), with relatively higher use in situations with unequal social status and intimate relationships. All other main effects and interaction effects were non-significant (Table 4; Fig 2).

For statement of obligation (Strategy 4), the main effect of S was statistically significant (*p* < 0.05, *eta²* = 0.0992, small-to-medium effect), with higher use among interlocutors of equal social status than those of unequal status. The interaction effect of R × D was statistically highly significant (*p* < 0.01, *eta²* = 0.1413, large effect), with the highest rate of use observed in situations with non-intimate relationships and difficult requests, and no statistically significant between-group differences in other contexts. All other main effects and interaction effects were non-significant (Table 4; Fig 2).

For make a suggestion (Strategy 5), only the interaction effect of S × D was statistically highly significant (*p* < 0.01, *eta²* = 0.1665, large effect). The highest rate of use was found in situations with unequal social status and easy requests, while the lowest rate was in situations with equal social status and easy requests. This result indicates that the combination of situational factors is the core influence on the use of this strategy, with a large substantive effect. All other main effects and interaction effects were non-significant (Table 4; Fig 2).

For express a wish (Strategy 6), only the main effect of D was statistically significant (*p* < 0.05, *eta²* = 0.0926, small-to-medium effect), with higher use in difficult situations than in easy ones. All other main effects and interaction effects were non-significant (Table 4; Fig 2).

For statement of the situation (Strategy 7), the main effect of P was statistically highly significant (*p* < 0.001, *eta²* = 0.5533, large effect), representing the most influential factor on the use of this strategy. NK used this strategy at a far lower rate than CKLA and CKLI, with no statistically significant difference between the latter two groups. The main effects of R (*p* < 0.01, *eta²* = 0.1424, large effect), S (*p* < 0.05, *eta²* = 0.1067, medium effect), and D (*p* < 0.05, *eta²* = 0.0813, small-to-medium effect) were also all statistically significant. Specifically, the use of this strategy was significantly higher with interlocutors of unequal social status, non-intimate interlocutors, and in difficult situations. All interaction effects were non-significant, with corresponding *eta²* values below 0.05 (Table 4; Fig 2).

### Refusal speech acts and associated strategies

Based on the results of the one-way ANOVA and post-hoc comparisons of refusal speech acts extracted from the dialogues, the overall between-group difference in the number of refusal strategy categories used was statistically highly significant (*p* < 0.001, *eta²* = 0.2288, large effect). Post-hoc comparisons showed no statistically significant difference between NK and CKLA in the number of refusal strategy categories used (*p* = 0.2853, Cohen’s *d* = 0.2718, small effect). In contrast, NK used a significantly greater variety of refusal strategies than CKLI (*p* < 0.0001, Cohen’s *d* = 1.1843, large effect), and CKLA also used significantly more refusal strategy categories than CKLI (*p* = 0.0017, Cohen’s *d* = 1.2480, large effect) (Table 3; Fig 1).

For the total number of refusal strategies used in the dialogue, the overall between-group difference was also statistically highly significant (*p* < 0.001, *eta²* = 0.3615, large effect). Post-hoc comparisons revealed no statistically significant difference between NK and CKLA (*p* = 0.9280, Cohen’s *d* = 0.0230, negligible effect), while NK used a significantly greater number of refusal strategies than CKLI (*p* < 0.0001, Cohen’s *d* = 1.6089, large effect), and CKLA also used significantly more refusal strategies than CKLI (*p* < 0.0001, Cohen’s *d* = 1.7987, large effect) (Table 3; Fig 1).

A direct comparison of effect sizes between groups provides explicit empirical support for the relative gap in language proficiency. the Cohen’s *d* values for comparisons between NK and CKLA were consistently at the small effect level or lower, while the Cohen’s *d* values for comparisons between NK and CKLI all reached the large effect level. This result confirms that the language proficiency and pragmatic competence gap between NK and CKLA is substantially smaller than the gap between NK and CKLI, and that higher Korean proficiency is associated with greater convergence in refusal speech act strategy use with native speakers.

In terms of the proportional use of specific refusal strategies, there was a statistically significant overall between-group difference in the use of provide a reason or rationale (Strategy 2) (*p* < 0.001, *eta²* = 0.2035, large effect). Post-hoc comparisons showed that NK used this strategy at a significantly lower rate than CKLA (*p* = 0.0149, Cohen’s *d* = 0.7265, medium effect), and at a significantly lower rate than CKLI (*p* < 0.0001, Cohen’s *d* = 1.1954, large effect). This finding indicates that overuse of this strategy is a core pragmatic difference between Chinese Korean language learners and native speakers, with the difference being more pronounced in intermediate learners (Table 3; Fig 2).

For the use of avoidance (Strategy 3), the overall between-group difference was statistically highly significant (*p* < 0.001, *eta²* = 0.1871, large effect). NK used this strategy at a significantly higher percentage than CKLA (*p* = 0.0007, Cohen’s *d* = 0.9021, large effect), and at a significantly higher percentage than CKLI (*p* = 0.0013, Cohen’s *d* = 0.8234, large effect). There was no statistically significant difference between CKLA and CKLI (*p* = 0.8438, Cohen’s *d* = 0.0987, negligible effect), indicating that underuse of the avoidance strategy is a consistent pragmatic feature of Chinese Korean language learners that does not change significantly with increasing language proficiency (Table 3; Fig 2).

For the use of accusation (Strategy 6), the overall between-group difference did not reach statistical significance (*p* > 0.05, *eta²* = 0.0733, medium effect). However, post-hoc tests revealed that CKLA used this strategy at a significantly higher rate than CKLI (*p* = 0.0224, Cohen’s *d* = 1.0035, large effect), with no other statistically significant pairwise comparisons (Table 3; Fig 2).

No statistically significant overall between-group differences were found for the use of direct refusal (Strategy 1), conditional acceptance (Strategy 4), subjective causes (Strategy 5), and express emotions (Strategy 7) (all *p* > 0.05), with all corresponding *eta²* values below 0.05 (small effect), indicating no substantive pragmatic differences in the use of these strategies across the three groups (Table 3).

Overall, differences in language proficiency were the core factor driving the divergence in the number of strategy categories, total number of strategies, and specific strategy choices between the three groups in the request-refusal continuous dialogue. The differences between NK and CKLI were the most prominent, with corresponding effect sizes consistently reaching the medium level or above, while the strategy use features of CKLA were highly convergent with those of NK, with only small effect levels for between-group differences.

A multifactor ANOVA was further conducted to analyze the effects of P, S, R, D, and their interactions on refusal speech acts in multi-round dialogues across different scenarios, with detailed results presented in Table 5.

**Table 5:** Results of multi-factor ANOVA examining the effects of target people (P), social status (S), intimacy of the relationship (R), difficulty (D), and their interaction on refusal speech act strategy use.

	Strategy category	Strategy number	Strategy 1	Strategy 2	Strategy 3	Strategy 4	Strategy 5	Strategy 6	Strategy 7
P	10.0358 ^***^ (0.2949)	20.9773 ^***^ (0.4664)	0.9907 ^ns^ (0.0396)	8.6541 ^***^ (0.2650)	9.8259 ^***^ (0.2905)	0.4870 ^ns^ 0.0199	0.5904 ^ns^ (0.0240)	3.7396 ^*^ (0.1348)	0.0803 ^ns^ (0.0033)
S	0.5936 ^ns^ (0.0122)	2.3669 ^ns^ (0.0470)	0.8823 ^ns^ (0.0180)	0.9389 ^ns^ (0.0192)	2.3461 ^ns^ (0.0466)	3.1149 ^ns^ (0.0609)	0.3658 ^ns^ (0.0076)	0.0000^ns^ (0.0000)	1.9551 ^ns^ (0.0391)
R	2.2013 ^ns^ (0.0439)	7.7550 ^**^ (0.1391)	0.7692 ^ns^ (0.0158)	2.2769 ^ns^ (0.0453)	0.5304 ^ns^ (0.0109)	1.0445 ^ns^ (0.0213)	0.0088 ^ns^ (0.0002)	0.3766 ^ns^ (0.0078)	10.3303 ^**^ (0.1771)
D	0.4922 ^ns^ (0.0102)	5.9232 ^*^ (0.1098)	0.0468 ^ns^ (0.0010)	1.5317 ^ns^ (0.0309)	2.0784 ^ns^ (0.0415)	1.2205 ^ns^ (0.0248)	4.1893 ^*^ (0.0803)	7.5591 ^**^ (0.1361)	6.2415 ^*^ (0.1151)
P × S	0.3852 ^ns^ (0.0158)	0.0533 ^ns^ (0.0022)	0.3768 ^ns^ (0.0155)	0.2787 ^ns^ (0.0115)	0.5023 ^ns^ (0.0205)	0.3714 ^ns^ (0.0152)	0.8199 ^ns^ (0.0330)	1.5469 ^ns^ (0.0605)	0.1868 ^ns^ (0.0077)
P × R	0.2855 ^ns^ (0.0118)	0.5026 ^ns^ (0.0205)	0.4158 ^ns^ (0.0170)	0.2845 ^ns^ (0.0117)	0.1465 ^ns^ (0.0061)	0.5870 ^ns^ (0.0239)	0.1794 ^ns^ (0.0074)	4.3127 ^*^ (0.1523)	0.1872 ^ns^ (0.0077)
P × D	3.0482 ^ns^ (0.1127)	0.8511 ^ns^ (0.0342)	0.5529 ^ns^ (0.0225)	0.0449 ^ns^ (0.0019)	6.1144 ^**^ (0.2030)	0.5272 ^ns^ (0.0215)	2.0895 ^ns^ (0.0801)	1.0680 ^ns^ (0.0426)	0.9209 ^ns^ (0.0370)
S × R	0.4300 ^ns^ (0.0089)	0.0231 ^ns^ (0.0005)	0.4918 ^ns^ (0.0101)	7.7575 ^**^ (0.1391)	0.0000 ^ns^ (0.0000)	2.8911 ^ns^ (0.0568)	6.0704 ^*^ (0.1123)	6.1284 ^*^ (0.1132)	0.0964 ^ns^ (0.0020)
S × D	1.0455 ^ns^ (0.0213)	0.3698 ^ns^ (0.0076)	0.8313 ^ns^ (0.0170)	0.4268 ^ns^ (0.0088)	5.5218 ^*^ (0.1032)	0.1506 ^ns^ (0.0031)	0.0307 ^ns^ (0.0006)	0.7224 ^ns^ (0.0148)	6.2070 ^*^ (0.1145)
R × D	4.3114 ^*^ (0.0824)	4.9241 ^*^ (0.0930)	0.1306 ^ns^ (0.0027)	1.2536 ^ns^ (0.0255)	0.3498 ^ns^ (0.0072)	0.6270 ^ns^ (0.0129)	2.5168 ^ns^ (0.0498)	0.6600 ^ns^ (0.0136)	0.9286 ^ns^ (0.0190)
P × S × R	0.0047 ^ns^ (0.0002)	0.1125 ^ns^ (0.0047)	0.5639 ^ns^ (0.0230)	0.4097 ^ns^ (0.0168)	0.4721 ^ns^ (0.0193)	0.1772 ^ns^ (0.0073)	0.4435 ^ns^ (0.0181)	2.0177 ^ns^ (0.0775)	0.3194 ^ns^ (0.0131)
P × S × D	0.5841 ^ns^ (0.0238)	0.0289 ^ns^ (0.0012)	0.7022 ^ns^ (0.0284)	0.0498 ^ns^ (0.0021)	5.0631 ^*^ (0.1742)	0.7658 ^ns^ (0.0309)	0.4225 ^ns^ (0.0173)	1.7874 ^ns^ (0.0693)	1.5721 ^ns^ (0.0615)
P × R × D	0.5683 ^ns^ (0.0231)	0.4942 ^ns^ (0.0202)	0.3106 ^ns^ (0.0128)	1.1916 ^ns^ (0.0473)	0.3576 ^ns^ (0.0147)	0.7501 ^ns^ (0.0303)	0.0052 ^ns^ (0.0002)	2.1243 ^ns^ (0.0813)	0.6090 ^ns^ (0.0247)
S × R × D	0.3843 ^ns^ (0.0079)	0.0124 ^ns^ (0.0003)	0.6449 ^ns^ (0.0133)	0.0302 ^ns^ (0.0006)	1.0720 ^ns^ (0.0218)	3.6035 ^ns^ (0.0698)	0.1807 ^ns^ (0.0038)	3.6187 ^ns^ (0.0701)	12.0039 ^**^ (0.2001)
P × S × R × D	0.2281 ^ns^ (0.0094)	0.3161 ^ns^ (0.0130)	1.1282 ^ns^ (0.0449)	0.5005 ^ns^ (0.0204)	0.0845 ^ns^ (0.0035)	0.2308 ^ns^ (0.0095)	0.3818 ^ns^ (0.0157)	0.8982 ^ns^ (0.0361)	0.1148 ^ns^ (0.0048)

Groups include NK, CKLA, and CKLI; social status includes equal and unequal; relationship includes intimate and not intimate; difficulty includes easy and difficult. *F* and (*eta*^*2*^) values are given. ^***^ indicates *P* < 0.001, ^**^ indicates *P* < 0.01, ^*^ indicates *P* < 0.05, and ^ns^ indicates no statistically significant difference. *Eta*^*2*^ values are given in parentheses. Explanation of the definitions for strategy statistics can be found in Data analysis methods.

For the number of refusal strategy categories used, the main effect of P was statistically highly significant (*p* < 0.001, *eta²* = 0.2949, large effect), representing the core factor influencing the diversity of refusal strategy use, with far greater explanatory power than individual situational factors. The interaction effect of R × D was statistically significant (*p* < 0.05, *eta²* = 0.0824, small-to-medium effect). Specifically, in difficult refusal situations, the most categories of refusal strategies were used with intimate interlocutors, while the fewest categories were used with non-intimate interlocutors. This result indicates that the combination of situational factors exerts a modest substantive effect on the diversity of refusal strategy use. All other main effects and interaction effects were non-significant, with corresponding *eta²* values below 0.05 (small effect) (Table 5; Fig 1).

For the total number of refusal strategies used, the main effect of P was statistically highly significant (*p* < 0.001, *eta²* = 0.4664, large effect), representing the decisive factor influencing the frequency of refusal strategy use. The main effects of R (*p* < 0.01, *eta²* = 0.1391, large effect) and D (*p* < 0.05, *eta²* = 0.1098, medium effect) were also statistically significant. Specifically, a greater number of refusal strategies were used with intimate interlocutors than with non-intimate ones, and more strategies were used in easy refusal situations than in difficult ones. Meanwhile, the interaction effect of R × D was statistically significant (*p* < 0.05, *eta²* = 0.0930, small-to-medium effect), with a relatively high number of refusal strategies used in contexts with intimate relationships, and the lowest number used in contexts with non-intimate relationships and easy refusal tasks. All other main effects and interaction effects were non-significant, with corresponding *eta²* values below 0.05 (Table 5; Fig 1).

For direct refusal (Strategy 1) and conditional acceptance (Strategy 4), none of the main effects or interaction effects reached statistical significance, with all corresponding *eta²* values below 0.05 (small effect), indicating that neither target group nor situational factors exerted a substantive influence on the use of these two strategies (Table 5; Fig 2).

For provide a reason or rationale (Strategy 2), the main effect of P was statistically highly significant (*p* < 0.001, *eta²* = 0.2650, large effect), the core factor influencing the use of this strategy, with NK using it at a significantly lower rate than Chinese Korean language learners. Meanwhile, the interaction effect of S × R was also statistically highly significant (*p* < 0.01, *eta²* = 0.1391, large effect), with the highest rate of use observed in situations with unequal social status and non-intimate relationships, and no statistically significant differences in other contexts. All other main effects and interaction effects were non-significant (Table 5; Fig 2).

For avoidance (Strategy 3), the main effect of P was statistically highly significant (*p* < 0.001, *eta²* = 0.2905, large effect), with NK using this strategy at a significantly higher rate than CKLA and CKLI. Meanwhile, the interaction effect of P × D was statistically highly significant (*p* < 0.01, *eta²* = 0.2030, large effect). Specifically, NK used the avoidance strategy at a higher rate in easy refusal situations and a lower rate in difficult situations; CKLI showed the same trend as NK, while CKLA showed no statistically significant difference in use across difficulty levels. In addition, the interaction effect of S × D (*p* < 0.05, *eta²* = 0.1032, medium effect) and the interaction effect of P × S × D (*p* < 0.05, *eta²* = 0.1742, large effect) were also statistically significant. These results indicate that the combination of situational factors exerts a large, substantive effect on the use of this strategy, and that this effect differs statistically significantly across participants with different language proficiency levels (Table 5; Fig 2).

For subjective causes (Strategy 5), only the main effect of D was statistically significant (*p* < 0.05, *eta²* = 0.0803, small-to-medium effect), with higher use in difficult refusal situations than in easy ones. The interaction effect of S × R was also statistically significant (*p* < 0.05, *eta²* = 0.1123, medium effect). Specifically, the use of this strategy was higher when refusing intimate interlocutors of equal social status than when refusing non-intimate interlocutors of equal social status; conversely, it was lower when refusing intimate interlocutors of unequal social status than when refusing non-intimate interlocutors of unequal social status. All other main effects and interaction effects were non-significant (Table 5; Fig 2).

For accusation (Strategy 6), the main effect of P was statistically significant (*p* < 0.05, *eta²* = 0.1348, large effect), as was the main effect of D (*p* < 0.01, *eta²* = 0.1361, large effect), with higher use in difficult refusal situations than in easy ones. Meanwhile, the interaction effect of P × R (*p* < 0.05, *eta²* = 0.1523, large effect) and the interaction effect of S × R (*p* < 0.05, *eta²* = 0.1132, medium effect) were also statistically significant. Specifically, NK used the accusation strategy at a higher rate when refusing intimate interlocutors than non-intimate ones, while CKLA showed the opposite trend, and CKLI showed no statistically significant difference across relationship intimacy levels. The lowest rate of use across all participants was observed in situations with equal social status and non-intimate relationships. All other main effects and interaction effects were non-significant (Table 5; Fig 2).

For express emotions (Strategy 7), the main effects of R (*p* < 0.01, *eta²* = 0.1771, large effect) and D (*p* < 0.05, *eta²* = 0.1151, medium effect) were statistically significant. This strategy was used at a higher rate with non-intimate interlocutors than with intimate ones, and at a higher rate in easy refusal situations than in difficult ones. Meanwhile, the interaction effect of S × D (*p* < 0.05, *eta²* = 0.1145, medium effect) and the interaction effect of S × R × D (*p* < 0.01, *eta²* = 0.2001, large effect) were also statistically highly significant. The highest rate of use was found in situations with unequal social status, non-intimate relationships, and easy refusal tasks, while the lowest rate was in situations with equal social status, intimate relationships, and difficult refusal tasks. All other main effects and interaction effects were non-significant (Table 5; Fig 2).

## Discussion

In daily communication, people frequently make requests, which may be either accepted or refused. As a responsive act, refusal naturally occurs in conjunction with requests. Both requests and refusals are face-threatening behaviors. The reality of how to make requests appropriately and how to refuse them in a way that does not damage the face of the other party to the greatest extent possible is inescapable. To be able to achieve their final goals, both the party making the request and the party refusing the request will adjust their polite expression strategies according to various realities, such as the level of status, the closeness of the relationship, the difficulty of the matter, etc., to ease the conflict to the greatest extent possible. The use of request and refusal strategies in dialogue is in a dynamic state of flux as the level of interaction between the parties varies and will determine the outcome of the communication. Much of the research that has been done on requests and refusals tends to study only one speech act, either request or refusal, in isolation, with very few studies of the two speech acts together as a whole [13,29–31]. Neglecting the correlation between request and refusal and separating the two for research, the results obtained are not in-depth, and comprehensive. Only by considering the request-refusal speech act as a continuous discourse process, and conducting quantitative research on the successive rounds of dialogues, can we comprehensively reveal the characteristics of the request-rejection dialogue act, explore the factors affecting its changes, and use them to guide the practice of foreign language teaching.

It was found that factors such as age, social power, social distance, the purpose of the request, the scenarios, the relationship, and the burden of the request influenced request behavior [23]. The influence of linguistic and cultural diversity on the extent of request speech acts, as well as the specific criteria for analyzing request speech act strategies, have been studied and discussed by numerous scholars. According to the analytical framework of the Cross-Cultural Speech Act Realisation Project (CCSARP), which has been proven to be effective, request speech acts were classified into three main strategies, and nine sub-strategies were proposed. This analytical framework has been applied in many subsequent request behavior studies [5,23]. Most studies on Korean request discourse refer to CCSARP’s analytical framework. Among the many studies on Korean request speech acts, the influencing factors involved are the attributes of the request, the content of the request, relationship, social status, age, gender [7,9–13,29,30], etc.

Refusal strategies are considered direct or indirect linguistic formulas. Specifically, which refusal strategy is used depends on the social status and power of both speakers. The order of use, frequency of occurrence, and specific content of linguistic formulas have also been analyzed. The types of refusal have been classified into direct and indirect refusals, and direct refusals have been further classified into performative and non-performative refusals. Indirect refusals were further categorized into 11 types [15], which has been the basis for several subsequent studies. Social status, closeness, task difficulty, and demands/requests, were identified as the four factors determining the degree of linguistic indirectness, which influenced the choice of variable factors in many subsequent studies [32]. In the study of refusal behavior in the Korean language, social status, social rights, social distance, the difficulty of the event, intimacy, gender, and age are among the influences that have been studied [4,13,16–18,20–22,29,30,33].

Although there are many influence factors for the strategic choice of request and refusal speech acts, both have some major common factors, such as social status, difficulty, and intimacy. Therefore, these can be identified as influence factors in the study of request-refusal continuous dialogue. Depending on the level of refinement of the analysis, there are up to a dozen different sub-strategies for request and refusal strategies, respectively. These differences are due to the different classification criteria and levels of refinement used in each study, as well as the different purposes and audiences of the studies. In foreign language learning activities, teachers do not have a high level of language proficiency in their target audience, especially beginner learners, so detailed categorization, while academically meaningful, may hinder rather than help learners select and use strategies in actual interactions. Therefore, the common influence factors, as well as the optimized request and refusal strategy analysis framework, can be used as a basis for the questionnaire involving the use of MICMF.

The results of the study showed that there were significant differences between NK and CKL in the choice of request and refusal dialogue strategies, with the most pronounced differences between NK and CKLI. For each group, the level of intimacy between interlocutors, social status, and difficulty of the speech act, and the interaction of these factors, may influence the choice of strategy. For example, there was a high use of request strategy 2 with intimate relationships and a relatively low use of request strategy 2 in scenarios where social status is unequal, and requests are easy to make. A higher proportion used the refusal strategy 6 in situations where refusal was difficult. NK used refusal strategy 6 more often when refusing an intimate person than when refusing a person who was not intimate.

A certain level of linguistic competence is both a prerequisite and a means of discursive expression. Request and refusal are demanding and politeness expression speech acts with a certain potential for face-threatening in their nature. Therefore, there is a gap between second language learners with limited knowledge of discourse language and socio-pragmatics and native speakers in their use [14]. According to the results of the statistical analyses, CKLA used a variety of strategies that exhibited transitional characteristics. Although CKLA and NK’s strategy choices were similar in many ways, it was difficult to distinguish between CKLA and CKLI in the use of some strategies in the statistical data.

The role of context is highly valued in pragmatics. Speakers should first have appropriate linguistic-pragmatic knowledge, and in combination with a specific context, use their socio-pragmatic knowledge to choose an appropriate way of expressing verbal behavior to achieve their intentions.

In the teaching of a second language, to achieve good results, the first step is to understand the students’ current language level, their habits of expression, and where the main gaps are between them and native speakers. The MICMF can be used to investigate the level of awareness of language behaviors of learners and native speakers. The statistical results of the data in this thesis are slightly different from the results of previous research dissertations [13], indicating that for different groups and sizes of samples, there will be some differences in language proficiency. Therefore, a pre-survey needs to be conducted for each group of students. More relevant data should be collected from different groups of native speakers as a standard of authentic expression in teaching. According to the results of the survey and analysis, second language learners should be taught according to their abilities and levels. Situating the use of speech acts makes it easier for learners to understand appropriate expressions in different complex situations. Narrowing the gap between learners’ and native speakers’ pragmatic competence in different contexts.

## Conclusions

With the methodology of MICMF, different variables regarding request and refusal communication strategies were investigated. Each variable was cross-combined with each other, and each combination was set up with three different repetitive scenarios, for a total of 24 consecutive dialogue request-refusal scenarios. The optimized classification of each into seven categories of request and refusal strategies was used as an analysis framework. DCT was combined with role-playing methods to obtain analytical data. Due to the different scenarios, it was appropriate to use two to three categories and two to four numbers in the request strategy. For refusal strategies, two to three categories, and anywhere from two to five (with three or four being appropriate). There were statistically significant differences in the frequency of use of request and refusal strategies across scenarios for NK, CKLA, and CKLI. Overall, NK used more categories and numbers of strategies than CKLI. The gap between NK and CKL may narrow as the language level increases. Another important finding was that not only a single influence factor affected strategy choice but also the interaction of these factors, suggesting that people might behave differently when faced with complex scenarios consisting of multiple factors.

The research methodology of MICMF combines factors affecting strategy choice by inserting them into individual scenarios, designing multiple independent scenarios with the same factors, and analyzing the effects of these factors and their interactions on strategy choice. Using this approach, differences in strategy choice between foreign language learners and native speakers in different scenarios are identified by analyzing multi-component cross-cultivated language scenarios, and the results of the analyses are used to help formulate detailed teaching plans. Based on the analysis results, targeted teaching priorities are set for learners at different proficiency levels. For intermediate learners, who lag far behind native speakers in the variety and quantity of strategies used and lack pragmatic cognition of multi-factor interactions, teaching centers on consolidating the reserve of basic strategies, expanding the dimensions of strategy application, strengthening pragmatic cognition of social status and interpersonal intimacy, and conducting basic situational training with single variables. For advanced learners, who have a narrow gap with native speakers in strategy quantity yet show inadequate adaptability in complex scenarios and insufficient cultural cognition, the teaching focus lies in enhancing their ability to flexibly select strategies in multi-factor scenarios, deepening the interpretation of cultural differences, optimizing the frequency of strategy use, and carrying out cross-cultural communication practice. A diverse range of teaching methods such as corpus-based teaching and situational role-play can be adopted for both groups to boost their pragmatic competence.

It should also be objectively noted that this study was mainly conducted in Qingdao, China and South Korea, and the research samples had certain geographical limitations—only participants from the aforementioned regions were recruited for the investigation, which might restrict the generalizability and applicability of the study’s conclusions to a certain extent. Based on this, in subsequent research, the geographical coverage of research samples can be further expanded to include participants from various regions both domestically and internationally, thereby enhancing the representativeness and universality of the research results. At the same time, the research objects can be extended to groups of Korean language learners with different native language backgrounds to further enrich the system of research findings, which will endow the conclusions of this study on request-refusal speech acts and the proposed MICMF with greater generalizable value and promising prospects for practical application.
